# Reply to Comments: A Lightweight and Low-Power UAV-Borne Ground Penetrating Radar Design for Landmine Detection

**DOI:** 10.3390/s20103001

**Published:** 2020-05-25

**Authors:** Danijel Šipoš, Dušan Gleich

**Affiliations:** Faculty of Electrical Engineering and Computer Science, University of Maribor, Koroska Cesta 46, 2000 Maribor, Slovenia; dusan.gleich@um.si

In this brief note, we respond to the comments made by Dr. Álvarez regarding the claim of incomplete or incorrect comparison of [1,2], which can be found in Section 5.3 of [1]. After a careful analysis of the comments, the replies are as follow:

The first comment claims that the system of [2] is based on Synthetic Aperture Radar (SAR) processing, however the proposed system of [1] is not. The focus of [1] was, as already the title states, on a lightweight and low-power Ground Penetrating Radar (GPR) design which was optimized for UAV use. Therefore, the comparison in Section 5.3 of [1] was limited only to the GPR performance, and not expanded to a complete Unmanned Aerial Vehicle (UAV) and GPR solution. Furthermore, the possibility of SAR processing is not related to the radar hardware itself, but the ability of precise radar localization and further data processing. Since the main focus of our work was on GPR development and not data processing or localization, this comment is out of our scope.

A comment was provided regarding: (i) the comparison of the developed GPR, found in Table 2 of [1] and the GPR used in [2], as well as (ii) The statement found in [1] related to Table 2:

*“The radar module [29] of System 3 has lower power consumption, but as in the paper is stated an on-board computer (Raspberry Pi) is used to store acquired data. This furthermore increases the power consumption.”*

The comment which is related to (i) and (ii) is as follows:

*“However, this is not correct, as the system described in [2] uses the Raspberry micro-computer for sending the measurements in real-time and also for controlling the UAV flight (i.e., an additional UAV flight controller is not used nor required). Therefore, the statement of Table 2 of [1], “Power consumption of system 3: 2–3 W (only radar)”, is incorrect.”*

The authors in [1] interpreted that a separate device (Raspberry Pi) was used only for data capturing due to the following statement found in [2]:

*“Flight control subsystem, which consists of a micro-computer (Raspberry Pi), a UAV flight controller and common positioning sensors (IMU, barometer, GNSS).”*

Since in the cited paragraph the micro-computer and UAV flight controller are provided separately, this has led to the misunderstanding.

Additionally, the provided comment includes that the statement in Table 2 of [1], where it was provided that only the radar device of [2] consumes 2–3 W is incorrect. In [2] it is stated that the PulsOn${}^{®}$ 440 radar module was used. Figure 16 from the manufacturer datasheet of the PulsOn${}^{®}$ 440 [3] device reveals that the consumption in Table 2 of [1] is indeed correct, since it is clearly related only to the radar which is not a standalone device.

The third comment is related to the scene overview of different systems stated in [1], and is the following:

*“This statement is not correct. As it can be checked in [2], tests with buried plastic objects have been conducted. In particular, Figure 11 of [2] shows the 18 cm diameter plastic disk that was buried in sand, as well as the SAR images taken using the airborne GPR, where the plastic disk is clearly visible. As stated in [2]:”*

The authors agree with the comment, as there is a missing indication that also plastic mines have been included in the measurements of [2]. However, this does not affect the comparison, since the scene overview has been provided only for a better insight.
